# Intraoperative Hyperglycemia during Liver Resection: Predictors and Association with the Extent of Hepatocytes Injury

**DOI:** 10.1371/journal.pone.0109120

**Published:** 2014-10-08

**Authors:** Sangbin Han, Justin Sangwook Ko, Sang-Man Jin, Hyo-Won Park, Jong Man Kim, Jae-Won Joh, Gaabsoo Kim, Soo Joo Choi

**Affiliations:** 1 Department of Anesthesiology and Pain Medicine, Samsung Medical Center, Sungkyunkwan University School of Medicine, Seoul, Korea; 2 Division of Endocrinology and Metabolism, Department of Medicine, Samsung Medical Center, Sungkyunkwan University School of Medicine, Seoul, Korea; 3 Department of Surgery, Samsung Medical Center, Sungkyunkwan University School of Medicine, Seoul, Korea; The University of Hong Kong, Hong Kong

## Abstract

**Background:**

Patients undergoing liver resection are at risk for intraoperative hyperglycemia and acute hyperglycemia is known to induce hepatocytes injury. Thus, we aimed to evaluate whether intraoperative hyperglycemia during liver resection is associated with the extent of hepatic injury.

**Methods:**

This 1 year retrospective observation consecutively enrolled 85 patients undergoing liver resection for hepatocellular carcinoma. Blood glucose concentrations were measured at predetermined time points including every start/end of intermittent hepatic inflow occlusion (IHIO) *via* arterial blood analysis. Postoperative transaminase concentrations were used as surrogate parameters indicating the extent of surgery-related acute hepatocytes injury.

**Results:**

Thirty (35.5%) patients developed hyperglycemia (blood glucose > 180 mg/dl) during surgery. Prolonged (≥ 3 rounds) IHIO (odds ratio [OR] 7.34, *P* = 0.004) was determined as a risk factors for hyperglycemia as well as cirrhosis (OR 4.07, *P* = 0.022), lower prothrombin time (OR 0.01, *P* = 0.025), and greater total cholesterol level (OR 1.04, *P* = 0.003). Hyperglycemia was independently associated with perioperative increase in transaminase concentrations (aspartate transaminase, β 105.1, standard error 41.7, *P* = 0.014; alanine transaminase, β 81.6, standard error 38.1, *P* = 0.035). Of note, blood glucose > 160 or 140 mg/dl was not associated with postoperative transaminase concentrations.

**Conclusions:**

Hyperglycemia during liver resection might be associated with the extent of hepatocytes injury. It would be rational to maintain blood glucose concentration < 180 mg/dl throughout the surgery in consideration of parenchymal disease, coagulation status, lipid profile, and the cumulative hepatic ischemia in patients undergoing liver resection for hepatocellular carcinoma.

## Introduction

The liver is one of principal organs for glycemic homoeostasis. Accordingly, patients undergoing liver resection are at risk of hyperglycemia due to procedures incurring the liver and functional hepatic tissue loss [1]. Since the landmark study by Van den Berghe et al.[2], numerous studies have evaluated the clinical importance of hyperglycemia. While the first movement has arisen in the field of critical care medicine and cardiac surgery [3]–[5], studies in non-cardiac surgeries have followed thereafter [6]–[8]. Accumulating evidence suggests that the effect of perioperative hyperglycemia vary by surgery and respective importance of intra-/postoperative hyperglycemia is different [3], [4], [9].

In liver resection, few studies have evaluated the effects of perioperative hyperglycemia mainly focusing on the extent of hepatic injury. It was reported that postoperative glucose concentration was associated with the extent of hepatic injury in hepatectomized patients [10]. A recent animal study in rats has demonstrated that acute hyperglycemia for a transient period amplifies hepatic ischemia reperfusion injury, which suggests the importance of acute hyperglycemia which occurs during surgery when hepatic ischemia injury may be accompanied [11]. However, the relationship between intraoperative hyperglycemia and hepatic injury has never been assessed in clinical liver resection. Thus, the objectives of this study were: (i) to evaluate the contributors for hyperglycemia during liver resection and (ii) to assess the relationship between intraoperative hyperglycemia and hepatocytes injury.

As liver enzymes retained in hepatocytes, aspartate transaminase (AST) and alanine transaminase (ALT) are well-known surrogate parameters for structural injury of hepatocytes cell barrier or necrosis [10], [11]. Recent studies further suggested that perioperative changes in serum concentration of the enzymes are correlated with clinical outcomes like liver synthetic function, regeneration, and biliary complication [12]–[14]. Thus, we evaluated postoperative transaminase concentrations to estimate the extent of surgery-related hepatocytes injury.

## Methods

### Subjects

Eight-five patients who underwent liver resection for hepatocellular carcinoma (HCC) between July 2011 and June 2012 who met the inclusion criteria were enrolled into the study in a consecutive manner. Exclusion criteria included preexisting diabetes, recurrent tumor, previous abdominal surgery, emergency, conjoined surgery, and American Society of Anesthesiologists physical grade ≥ 3. All patients underwent the standardized preoperative evaluation for hepatectomy candidacy based on Child-Pugh class and indocyanine green clearance test. Extrahepatic metastases were evaluated by chest computed tomography, abdominal computed tomography/magnetic resonance imaging, technetium-99m bone imaging, and positron emission tomography-computed tomography. The Institutional Review Board of Samsung Medical Center approved this retrospective study (SMC 2014-05-115) and waived the requirement of written informed consent. All data were obtained from ordinary electronic medical chart and were anonymized and de-identified prior to analysis.

### Monitoring and anesthesia

Anesthetic management was standardized. After the standard anesthetic monitoring (peripheral capillary oxygen saturation, 3-lead electrocardiography, non-invasive arterial blood pressure), anesthesia was induced with thiopental sodium (5 mg/kg) and maintained with isoflurane. Remifentanil was infused intravenously at the rate of 0.05–0.20 mcg/kg/min according to hemodynamic responses. Ventilation was controlled to obtain a tidal volume of 8 ml/kg and to maintain normocapnea, and the fraction of inspired oxygen was maintained at 0.5. Normothermia was maintained using a warm blanket and a passive humidifier. Circulation was managed to achieve a target mean arterial pressure and pulse pressure variation of 70 mmHg and 12%, respectively [15], [16]. Compound sodium lactate was primarily administered to maintain body fluid homeostasis. Packed red blood cells transfusion was indicated when blood hemoglobin concentration was < 8.0 mg/dl.

### Surgical procedures

The extent of surgical resection was determined based on computed tomography and magnetic resonance imaging. A parenchymal resection was performed using an ultrasonic dissector and a bipolar coagulator without clamp crushing method. Intermittent hepatic inflow occlusion (IHIO) was applied to reduce blood loss during parenchymal resection whenever needed in an ‘on-demand’ fashion. One round of the maneuver consisted of 15 min clamping of the hepatoduodenal ligament using a vascular tourniquet and 5 min unclamping. While checking hemostasis of the parenchymal cut surface during each period of unclamping, the maneuver was repeated in a step-by-step fashion as long as bleeding was considered to be mainly originated from the hepatic inflow. Anatomical variation or complexity was not an indication for the maneuver as described elsewhere [13], [14]. Preconditioning or postconditioning strategy was not used around IHIO.

### Glycemic control protocol

Patients fasted overnight and 5% dextrose solution in normal saline was infused at a rate of 80 ml/hr during the fasting period. Oral carbohydrate supplements were not given before surgery. Blood glucose concentration was measured using an arterial blood gas/chemistry analysis (RAPIDLAB1265, SIEMENS, Berlin, FRG) immediately after the anesthetic induction and every 2 hours thereafter. During the study period, blood glucose concentrations were additionally measured at the start/end of every IHIO and the end/1 hour/2 hours of parenchymal resection using a hand-held point-of-care glucometer analysis (Precision PCx, MediSense Inc., Bedford, MA) of the arterial blood drop due to concern of IHIO-induced severe glycemic disturbances based on a recent report by Maeda H. et al [17]. Intensive insulin therapy was not used while regular insulin was considered when glucose concentrations were > 200 mg/dl in serial measurements. Glucose was considered when glucose concentration was < 80 mg/dl. Attending surgeons followed up patients after surgery and recorded complications.

### Study objectives and parameters

The primary objective was to determine independent contributors for intraoperative hyperglycemia and the association between intraoperative hyperglycemia and the extent of hepatocytes injury. Hyperglycemia was defined as when the peak glucose concentration was > 180 mg/dl according to recent guidelines [18]. The extent of hepatocytes injury was estimated with delta (D)-transaminase concentrations (the peak postoperative value minus preoperative value) [12], [13]. Analyzed variables were demographic, laboratory, histological, surgical, and anesthetic factors including age, waist circumference, alcohol intake, coagulation status, total cholesterol, tumor biology, liver parenchymal histology, hepatic ischemia time, liver resection range, and blood loss. Immoderate alcohol consumption was defined when mean daily alcohol intake > 40 g for male and 20 g for female [19]. Major resection was defined as the resection ≥ 2 segments [20]. Pathologic features were analyzed based on the biopsies evaluation made by expert liver pathologists. The secondary objective was to explore the relationship between intraoperative hyperglycemia and postoperative clinical outcome. Any deviation from the normal postoperative course was considered as complication according to the Clavien-Dindo classification [21]. In short, grade III-V complications were defined as follows: III, complication requiring surgical, endoscopic or radiological intervention with or without general anesthesia; IV, life-threatening complication necessitating intensive care unit management; V, death of a patient due to complication. Biliary complication included hyperbilirubinemia (when the peak postoperative total bilirubin concentration was > 4.0 mg/dl and preoperative concentration was < 1.2 mg/dl) [22], bile leakage (when it presented at drainage bag and bilirubin concentration was confirmed to be higher in drainage fluid than in blood) and biliary stricture. Respiratory complication included atelectasis, pleural effusion and pulmonary edema based on radiologic evidences. Surgical site infection included the superficial/deep incisional infection or organ/space infection according to the Centers for Disease Control definitions for surgical infection [23].

### Statistics

Data were analyzed using SPSS 19.0 (SPSS Inc, Chicago, IL). Continuous variables were described as mean ± standard deviation or median (25 percentile, 75 percentile) according to a probability distribution and analyzed using a t-test or Mann-Whitney U test, respectively. Categorical variables were described as frequency (%) and analyzed using chi-square test, logistic regression or Fisher’s exact test, as appropriate. Multivariate binary logistic regression analysis was used for determining independent factors contributing to hyperglycemia. Variables of *P* < 0.25 in univariate analyses were selectively entered into the multivariate binary logistic regression model [24]; the results were described as the odds ratio (OR) with 95% confidential interval (CI). Multiple linear regression analysis was used for determining the association between blood glucose concentrations and D-transaminase concentrations; the estimated regression coefficients (β) were described with standard errors (SE). A *P* value of < 0.05 was considered statistically significant.

## Results

All 85 patients were Child-Pugh class A and confirmed to have HCC by pathologists: primary etiologies were hepatitis B virus (n = 76, 89.4%), hepatitis C virus (n = 7, 8.2%) and alcohol (n = 2, 2.4%). Thirty-one (36.5%) patients were pathologically diagnosed to have cirrhosis. Although no patients had diagnosed diabetes, 11 patients showed preoperative fasting blood glucose ≥ 126 mg/dl. Thirty-nine patients (46%) underwent major resection (2 segments, n = 33; 3 segments, n = 3; 4 segments, n = 2) and 45 patients (53%) underwent at least 1 round IHIO during parenchymal resection. Four patients received packed red blood cells (2 packs, n = 3; 1 pack, n = 1). Two patients intravenously received 2 units of regular insulin for managing sustained hyperglycemia. There were no patients who developed hypoglycemia or received glucose. There were no patients who underwent cardiopulmonary resuscitation or died during surgery.

**Figure 1:**
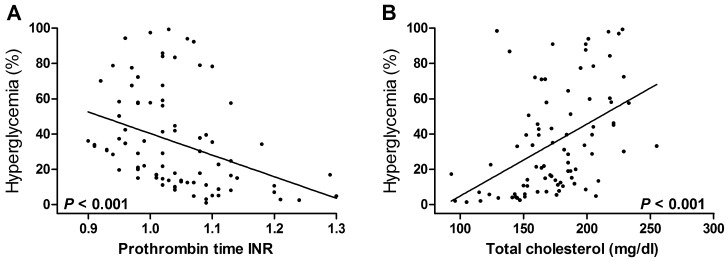
Probability of intraoperative hyperglycemia in relation to the increase in prothrombin time internationalized ratio and serum total cholesterol concentration.

Of 85, 30 (35.3%) patients developed hyperglycemia during surgery. As shown in Table 1, age, ALT, prothrombin time, creatinine, total cholesterol, IHIO time, cirrhosis, and tumor macrovascular invasion were potentially associated with hyperglycemia (*P* < 0.25). Mean age was greater in patients who developed hyperglycemia than in non-hyperglycemic patients (55 vs. 51 years). Serum ALT level was greater in non-hyperglycemic patients by trivial degree (37 vs. 30 mg/dl). Prothrombin time internationalized ratio (INR) was also greater in non-hyperglycemic patients (1.04 vs. 1.02). Serum total cholesterol level was significantly greater in hyperglycemic patients by around 20 mg/dl (186 vs. 168 mg/dl, *P* = 0.016). Serum creatinine was greater in hyperglycemic patients (0.93 vs. 0.89 mg/dl). IHIO ≥ 3 rounds was significantly associated with hyperglycemia (50.0% vs. 10.9%, *P* = 0.001) while 1–2 round IHIO was not (20.0% vs. 32.7%, *P* = 0.932). Cirrhosis was more common in hyperglycemic patients with a marginal significance (50.0% vs. 29.1%, *P* = 0.056). Tumor biological factors were not associated with hyperglycemia with the exception of macrovascular invasion (hyperglycemic 13.3% vs. non-hyperglycemic 1.8%, *P* = 0.050). Subsequent multivariate analysis confirmed that lower prothrombin time INR (OR 0.01, 95% CI 0.01–0.32), greater total cholesterol (OR 1.04, 95% CI 1.01–1.06), cirrhosis (OR 4.07, 95% CI 1.22–13.59), and ≥ 3 rounds IHIO (OR 7.34, 95% CI 1.90–28.39) were the independent risk factors for hyperglycemia (Table 2). Figure 1 showed the adjusted probability of hyperglycemia in relation to the increase in prothrombin time INR and total cholesterol level.

**Table 1:** Univariate analysis for intraoperative hyperglycemia during liver resection.

	Non-Hyperglycaemic (*n* = 55)	Hyperglycaemic (*n* = 30)	*P*
Age (year)	51.1 ± 10.6	55.0 ± 7.2	.080
Female gender	9 (16.4)	3 (10.0)	.527
Body mass index (kg/m^2^)	24.11 ± 2.86	24.38 ± 2.40	.665
Waist circumference	86.7 ± 8.4	86.7 ± 5.5	.372
Immoderate alcohol intake	11 (20.0)	9 (30.0)	.299
Never smoker	19 (34.5)	11 (36.7)	.845
Preoperative laboratory tests			
ICG15 > 10%	23 (41.8)	16 (53.3)	.309
Aspartate transaminase (mg/dl)	32 (25–40)	29 (20–59)	.516
Alanine transaminase (mg/dl)	37 (26–52)	30 (18–43)	.056
Total bilirubin (mg/dl)	0.6 (0.4–0.8)	0.6 (0.4–0.8)	.434
Prothrombin time (INR)	1.04 (0.99–1.10)	1.02 (0.98–1.06)	.065
Partial thromboplastin time (sec)	35.2 (33.6–37.0)	34.7 (33.4–36.0)	.369
Total cholesterol (mg/dl)	168 (147–188)	186 (162–211)	.016
Fasting glucose (mg/dl)	106 ± 20	105 ± 24	.716
Creatinine (mg/dl)	0.89 (0.80–0.97)	0.93 (0.84–1.07)	.120
Intermittent ischemia round			.001
0	31 (56.4)	9 (30.0)	
1–2 (vs. 0)	18 (32.7)	6 (20.0)	.932
≥ 3 (vs. 0)	6 (10.9)	15 (50.0)	.001
Major resection	25 (45.5)	14 (46.7)	.915
Histologic feature			
Liver cirrhosis	16 (29.1)	15 (50.0)	.056
Edmonson grade 3–4	10 (18.2)	5 (16.7)	.861
Tumor size (Cm)	4.0 ± 2.1	4.6 ± 3.0	.344
Tumor number ≥ 2	11 (20.0)	5 (16.7)	.707
Macrovascular invasion	1 (1.8)	4 (13.3)	.050
Bile duct invasion	1 (1.8)	0 (0)	>. 99
Mean arterial pressure (mmHg)	84 ± 9	83 ± 7	.696
Crystalloid replacement (ml/hr)	404 ± 107	407 ± 119	.896
Colloid replacement ≥ 1000 ml	5 (9.1)	4 (13.3)	.714
Estimated blood loss > 300 ml	10 (18.2)	3 (10.0)	.366
Operative time (hr)	4.2 ± 1.2	4.4 ± 1.3	.526
Minimum core temperature (°C)	35.7 ± 0.5	35.6 ± 0.4	.441

Data are presented as mean ± SD or number (%).

**Table 2:** Multivariate analysis for intraoperative hyperglycemia during liver resection.

	OR (95% CI)	*P*
Age (year)	1.03 (0.96–1.10)	.381
Alanine transaminase (mg/dl)	0.99 (0.98–1.01)	.374
Prothrombin time (INR)	0.01 (0.01–0.32)	.025
Creatinine (mg/dl)	2.11 (0.02–210.80)	.751
Total cholesterol (mg/dl)	1.04 (1.01–1.06)	.003
Intermittent ischemia round (vs. 0)		
1–2	0.96 (0.27–3.60)	.953
≥ 3	7.34 (1.90–28.39)	.004
Liver cirrhosis	4.07 (1.22–13.59)	.022
Macrovascular invasion of cancer	23.28 (0.47–1156.80)	.171

Due to concern of multicollinearity prothrombin time and total cholesterol level were separately enrolled into the multivariate model. Odds ratio and P values of other variables were described based on the model with prothrombin time internationalized ratio (INR).

**Figure 2:**
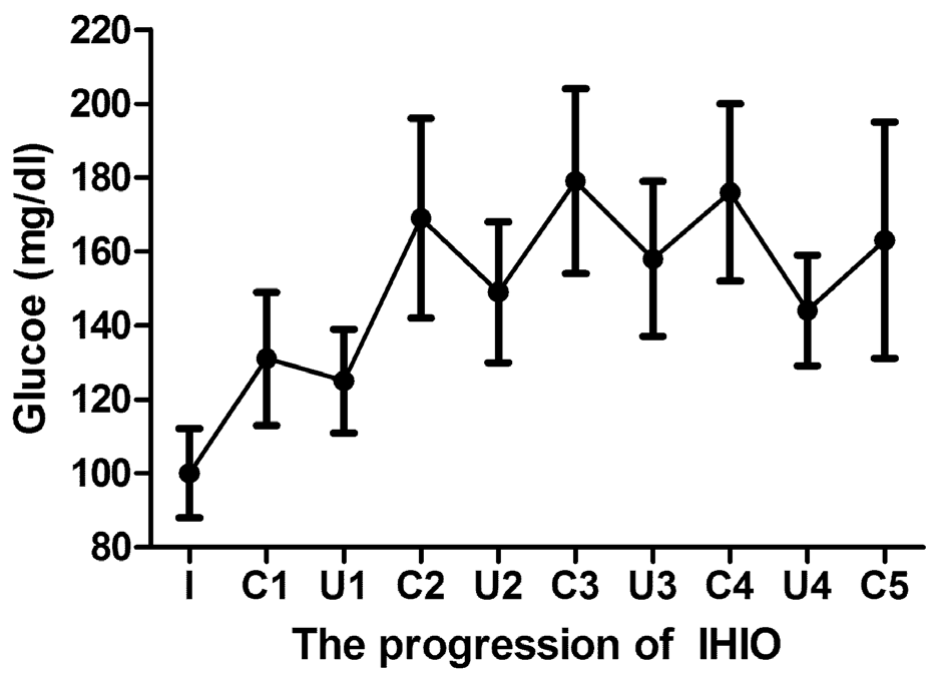
Up-and-down blood glucose fluctuation in response to clamping (C) and unclamping (U) of intermittent hepatic inflow occlusion (IHIO). I, anesthetic induction.

The detailed glycemic response of patients to IHIO is shown in Figure 2. Up-and-down glycemic fluctuation repeated in accordance with unclamping and clamping of IHIO. The degree of glucose rise for a 5 minutes reperfusion tended to decrease in progression: 40 ± 23, 30 ± 15, 17 ± 10, and 19 ± 19 mg/dl in accordance with 1st, 2nd, 3rd, and 4th IHIO.

**Figure 3:**
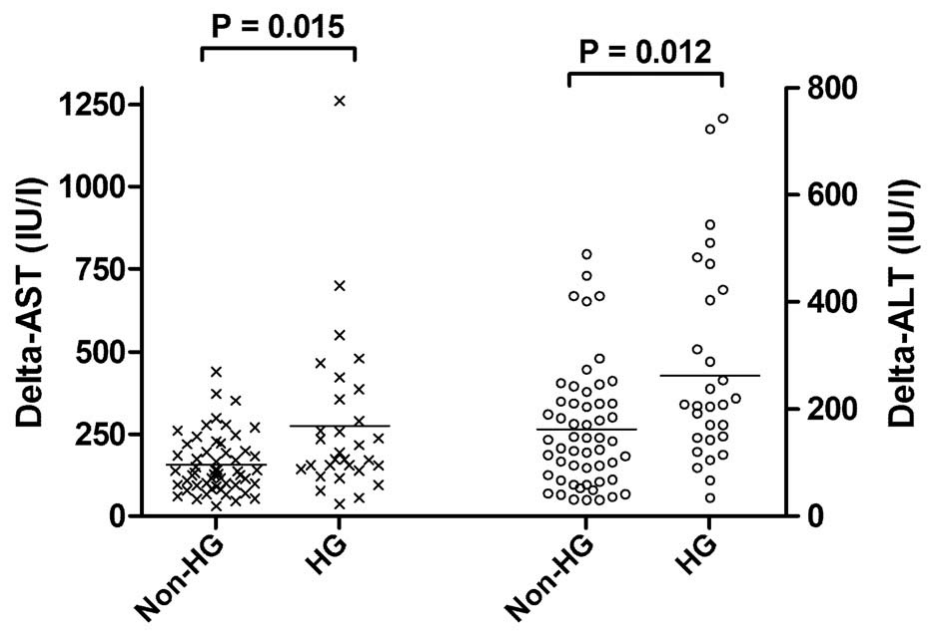
Significant difference in perioperative increase of AST (‘x’ mark) and ALT (‘o’ mark) according to the occurrence of intraoperative hyperglycemia (HG).

As shown in Figure 3, D-AST and D-ALT were significantly greater in patients who developed hyperglycemia than in non-hyperglycemic patients (157 ± 88 vs. 274 ± 243 IU/l, *P* = 0.015 and 162 ± 113 vs. 263 ± 193 IU/l, *P* = 0.012, respectively). Four hyperglycemic patients (13.3%) showed > 500 IU/l D-ALT, whereas no non-hyperglycemic patients showed such degree increase (*P* = 0.014). As shown in Table 3 and Table S1, sequent multivariate analyses confirmed that hyperglycemia was independently associated with greater D-AST (coefficient 104.9 ± 41.6, *P* = 0.014) and D-ALT (coefficient 81.1 ± 38.1, *P* = 0.036). In contrast, blood glucose concentration > 160 or 140 mg/dl had no significant impact on postoperative transaminase concentrations.

**Table 3:** Relationship between the peak intraoperative blood glucose concentrations and the peak postoperative transaminase concentrations.

	Delta-AST		Delta-ALT	
	β ± SE	*P*	β ± SE	*P*
> 140 mg/dl	49.3 ± 48.1	.309	55.9 ± 43.3	.201
> 160 mg/dl	67.0 ± 39.7	.095	33.1 ± 36.4	.381
> 180 mg/dl	104.9 ± 41.6	.014	81.1 ± 38.1	.036

*P* values were adjusted with confounders potentially associated with hepatocytes injury including age, gender, body mass index, presence of cirrhosis, tumor size, extent of surgery, and the total ischemia round. Delta-transaminase was calculated by the peak postoperative values minus preoperative value.

As shown in Table 4, postoperative clinical outcomes were not significantly different between non-hyperglycemic and hyperglycemic patients. Two hyperglycemic patients developed surgical site infection (infected biloma and peritonitis, respectively), whereas no control patients developed it. One hyperglycemic patient died of hepatic failure accompanied by multi-organ failure. The patient was non-cirrhotic and underwent extended left hemihepatectomy with 4 rounds IHIO (71 minutes cumulative ischemia). Tumor was 5 cm in size and showed macrovascular invasion upon both the hepatic vein and portal vein. The peak glucose concentration measured during surgery was 191 mg/dl.

**Table 4:** Postoperative outcomes of patients in the control and hyperglycaemic group.

	Non-hyperglycaemic (n = 55)	Hyperglycaemic (n = 30)	*P*
Overall complication	33 (60.0)	19 (63.3)	.763
Grade III-V complication	1 (1.8)	2 (6.7)	.283
Biliary complication	9 (16.4)	4 (13.3)	.711
Hyperbilirubinemia	6 (10.9)	1 (3.3)	.413
Bile leak	2 (3.6)	3 (10.0)	.340
Biliary stricture	1 (1.8)	2 (6.7)	.283
Transfusion	3 (5.5)	3 (10.0)	.661
Red blood cells	1 (1.8)	2 (6.7)	.661
Fresh frozen plasma	3 (5.5)	3 (10.0)	.283
Platelet	0	2 (40.0)	.444
Ileus	5 (9.1)	4 (13.3)	.714
Surgical site infection	0 (0.0)	2 (6.7)	.122
Respiratory complication	18 (32.7)	12 (40.0)	.503
Recurrence within 3 months	2 (3.6)	3 (10.0)	.340
Hospital stay (day)	14 ± 7	15 ± 7	.542
Hospital stay > 2 weeks	16 (29.1)	11 (36.7)	.473

Data are presented as number (%) or mean ± SD.

## Discussion

This study investigated blood glucose changes during liver resection in patients with chronic liver diseases and demonstrated three main findings. First, we affirmed the repeated up-and-down glucose fluctuation in response to intermittent hepatic ischemia and reperfusion. Second, we determined that prolonged IHIO (≥ 3 rounds or 45 minutes cumulative ischemia) increased the risk of intraoperative hyperglycemia along with cirrhosis, lower prothrombin time INR, and greater serum total cholesterol. Third, intraoperative hyperglycemia was associated with increased postoperative transaminase concentrations, indicating a greater hepatocytes injury.

The liver plays a prominent role for maintaining glucose metabolism. Thus, glycemic homeostasis might be disturbed in a condition of hepatic ischemia like clamping of the hepatic inflows. We observed acute hyperglycemic responses alternating with hypoglycemic responses in accordance with unclamping and clamping of the hepatic inflows, being in agreement with a recent study using a continuous glucose monitoring device in diseased patients undergoing liver resection [17]. The net effect of an up-and-down glycemic response leaned toward hyperglycemia, and accordingly, ≥ 3 rounds IHIO was associated with the increase in hyperglycemia risk. This phenomenon may share the mechanism with postreperfusion hyperglycemia shown immediately after graft reperfusion in liver transplantation [25]. Deprival of hepatic inflow decreases intracellular energy level and the delivery of glucose to the hepatocytes, leading to a massive glycogenolysis. Glucose cumulates inside hepatocytes and then leaks spontaneously or *via* necrosis. After hepatic reperfusion, substantial amounts of glucose retained in the liver flows into the systemic circulation and blood glucose concentration rises [26]. The graded decrease in glucose rise in the progression of IHIO could be explained by a graded decrease in liver cell stores of glycogen.

Apart from the extension of IHIO, baseline insulin sensitivity seemed to be important for the development of intraoperative hyperglycemia. Cirrhosis, lower prothrombin time, and greater total cholesterol level were determined as independent contributing factors for hyperglycemia. The relationship between chronic liver disease and insulin resistance was well-known. Hepatic parenchymal cell damage and replacement by fibrosis increases portal venous pressure and increase portal-systemic shunting. The delivery of insulin into hepatocytes is decreased and chronic hyperinsulinemia occurs together with inadequate beta cell response and peripheral/hepatic insulin resistance [27]. The significance contribution of prothrombin time INR and serum total cholesterol is supported by recent evidence suggesting interaction between visceral obesity, glucose/lipid metabolism, and blood coagulation [28]–[30]. Particularly, Kaji N. et al. clearly demonstrated an acceleration of blood coagulation and disturbance of lipid profile in insulin-resistant subjects by means of partial thromboplastin time, prothrombin time, total cholesterol, and triglyceride [31]. In our study, partial thromboplastin time was also shorter in hyperglycemic patients along with prothrombin time although a statistical significance was not obtained, possibly due to the lack of sufficient sample size to validate the trivial difference.

Hyperglycemia does not require prolonged exposure to exert its deleterious effects. Experimental research has shown that acute hyperglycemia for a transient period increased ischemia reperfusion injury of the liver as well as other organs and impaired microcirculation *via* oxidative stress and proinflammatory cytokines [1], [11], [32]–[34], which further impair insulin sensitivity and pancreatic insulin secretion [35]. Research in the clinical field also demonstrated the associations between intraoperative hyperglycemia and surgery-related infection and ischemia reperfusion injury in non-hepatic surgeries and liver transplantation [5], [7], [8]. Of importance, acute hyperglycemia abolished protective effects of ischemic preconditioning, which is an important theoretical benefit of IHIO [36], in cardiac surgery and experimental hepatectomy [11], [37]. A mechanistic study in critically ill patients by Vanhorebeek et al. has further demonstrated that hyperglycemia injures hepatocytes *via* impairment of the mitochondrial ultrastructure compartment and mitochondrial function [38]. Overall, it appeared that blood glucose concentrations greater than some degree have toxicity on hepatocytes. Despite accumulating evidence suggesting that acute hyperglycemia injures hepatocytes and increase the degree of ischemia reperfusion injury, there have been no study evaluating intraoperative hyperglycemia in patients undergoing liver resection who are at greater risk of glucose disturbances and might be more vulnerable to the hepatic effect of hyperglycemia. Few glycemic studies were found in clinical hepatectomy, however, they mainly focused on the postoperative period or intensive insulin therapy other than blood glucose concentration itself [10]. Other studies have only highlighted the applicability of intraoperative glycemic guidelines for liver resection on maintaining some target glucose ranges without considerations of hepatic effect of blood glucose concentrations [39]. Moreover, the above studies involved heterogeneous population consisting of patients with HCC and other primary/metastatic liver tumors.

The relationship between blood glucose concentration and hepatocytes injury was not continuously proportional. When blood glucose concentration was < 180 mg/dl, grade increase of blood glucose from < 140 mg/dl to > 140 mg/dl and > 160 mg/dl did not affect transaminase concentrations. In contrast, > 180 mg/dl glucose was significantly associated with transaminase rise. This finding suggested a liver tolerance of glucose toxicity, however, that might be imperfect and > 180 mg/dl glucose surpasses the tolerance and amplifies hepatocytes injury. This finding was in agreement with an animal study in rats which has demonstrated the tolerability of the liver to mild to moderate hyperglycemia regarding hepatocytes injury [11]. As mentioned above, acute hyperglycemia encompasses already-progressing hormonal changes/cell injuries and it is unlikely that returning blood glucose levels to within a normal range redresses all deleterious effects. Glycemic strategies specifically designed for liver resection using IHIO need to be developed based on avoidance strategy other than catch-up treatment.

Apart from hyperglycemia, there was a theoretical possibility of hypoglycemia. The liver was a primary site for insulin clearance *via* first-pass transit, and thus, reduction of liver mass in combination with reduced liver cell stores of glycogen and gluconeogenesis in chronic liver diseases raised concern for the development of hypoglycemia. However, an unlikelihood of hypoglycemia was found and the data suggested that glucose metabolism is well maintained in hepatectomized patients in agreement with a previous study demonstrating glycemic metabolism was well maintained after major hepatectomy [40]. This phenomenon might be attributable to the hypertrophy of counter part of tumors and consequent compensate of the metabolic capacity. Indeed, we applied a limited insulin use, based on 200 mg/dl in serial measurements, even compared to the standard glucose management based on 180 mg/dl [18], being mainly due to the uncertainty of insulin clearance/action during and after liver resection. Accordingly, only two patients received regular insulin during surgery. As might be expected from unlikelihood of hypoglycemia demonstrated in the present study, more intensive glucose management seemed to be feasible although this premise needs to be better validated in future studies. In terms of intensive insulin infusion, the standard glycemic management was thought to be reasonable in consideration of possible deleterious effects of hypoglycemia and liver tolerance to moderate hyperglycemia demonstrated in the present and other studies, being in line with recent guidelines suggesting 180 mg/dl as a standard [11], [18].

The following issues limit the applicability of the results of the present study. First, serum transaminase concentrations are widely used as sensitive and reliable parameters to evaluate the extent of surgery-related acute hepatocytes injury [11]–[13]. AST and ALT are enzymes present in hepatocytes and leak into the blood in the case of structural hepatocytes injury like necrosis. Although the applicability of the enzymes is limited in some cases at which surgical incursions to the liver is highly variable, it could be deduced that patients with and without intraoperative hyperglycemia were not significantly different regarding the surgical incursions. Surgical factors including the tumor size/invasion, extent of resection, parenchymal bleeding and operative time were comparable between the groups as well as anesthetic factors including hemodynamics, fluids replacement, and body core temperature. Nonetheless, histological evaluations and specific cytokines/proteases/reactive oxygen species would have contributed more scientifically to the cause-effect relationship between hyperglycemia and hepatocytes injury. Second, stress hormones induced by major surgery is known to result in insulin resistance by preventing insulin from suppressing hepatic gluconeogenesis and inducing glucose uptake into skeletal muscle [1]. Thus, the evaluation of plasma concentrations of stress hormones such as corticotropin, catecholamines, and growth hormone as well as insulin sensitivity and glucose effectiveness can help draw firmer conclusions in regard to the relationship between the cause of intraoperative hyperglycemia and hepatocytes injury and give mechanistic evidences. Third, there was a considerable possibility of type II error for secondary outcomes due to underpowered sample size. Given that hepatocytes injury is supposed to be related to regeneration of the injured liver including the biliary trees [13], it is warranted to focus on clinical outcomes in future research. Fourth, it could not be analyzed that how 11 patients with > 126 mg/dl preoperative fasting blood glucose affected the results of the study due to the insufficient size (Figure S1). This subgroup of patients with greater preoperative glucose level needs to be better evaluated in future studies. Fifth, primary etiology was HBV dominant. Mechanisms of glycemic disturbances are known to be different between chronic liver diseases originating from HBV, HCV, and alcohol [41]. Thus, the results of the present study might not translate into a different etiologic population.

In conclusion, the safe upper limit of blood glucose concentration was 180 mg/dl based on the extent of hepatocytes injury demonstrated by perioperative transaminase rise. Cirrhosis, lower prothrombin time, greater serum total cholesterol, and prolonged hepatic ischemia were determined as contributing factors for the development of intraoperative hyperglycemia. The information should help the selection or development of optimal glycemic management during liver resection for hepatocellular carcinoma.

## Supporting Information

Figure S1 **Changes in blood glucose concentration in response to clamping (C) and unclamping (U) of intermittent hepatic inflow occlusion (IHIO) of 11 patients with > 126 mg/dl preoperative glucose level. I, anesthetic induction.**
(TIF)

Table S1 **Multivariate analysis for intraoperative hyperglycemia during living donor right hepatectomy.**
(DOC)
