# Supplementary material for: Intraoperative Hyperglycemia during Liver Resection: Predictors and Association with the Extent of Hepatocytes Injury
Source: PLoS One. 2014 Oct 8;9(10):e109120. doi: 10.1371/journal.pone.0109120 (PMC4189957; doi:10.1371/journal.pone.0109120)
Supplement: Table S1 — Multivariate analysis for intraoperative hyperglycemia during living donor right hepatectomy. (DOC) [file pone.0109120.s002.doc]

**Supplementary Table 1** Multivariate analysis for intraoperative hyperglycemia during living donor right hepatectomy.

|  | OR (95% CI) | *P* |
| --- | --- | --- |
| Age (year)  Alanine transaminase (mg/dl)  Prothrombin time (INR)  Creatinine (mg/dl)  Total cholesterol (mg/dl)  Intermittent ischemia round (vs. 0)  1-2  ≥ 3  Liver cirrhosis  Macrovascular invasion of cancer | 1.02 (0.95-1.10)  0.99 (0.98-1.01)  0.01 (0.01-0.32)  35.17 (0.28-4426.60)  1.04 (1.01-1.06)  0.84 (0.21-3.32)  7.98 (1.92-33.18)  3.45 (1.01-11.73)  22.17 (0.44-1129.97) | .573  .292  .025  .149  .003  .802  .004  .048  .122 |

Due to concern of multicollinearity prothrombin time and total cholesterol level were separately enrolled into the multivariate model. Odds ratio and P values of other variables were described based on the model with total cholesterol.
